# Supplementary material for: Autonomous underwater adhesion driven by water-induced interfacial rearrangement
Source: Nat Commun. 2023 Oct 17;14:6563. doi: 10.1038/s41467-023-42209-2 (PMC10582181; doi:10.1038/s41467-023-42209-2)
Supplement: Supplementary file 1 — Supplementary Information [file 41467_2023_42209_MOESM1_ESM.pdf]

Supplementary Information

Autonomous underwater adhesion driven by water-induced interfacial rearrangement

Le Yao[1], Chengjiang Lin[2], Xiaozheng Duan[2]*, Xiaoqing Ming[1], Zhixuan Chen[1], He Zhu[1], Shiping Zhu[1], Qi Zhang[1]*

[1]School of Science and Engineering, The Chinese University of Hong Kong, Shenzhen, Shenzhen, Guangdong 518172, P.R. China. [2]State Key Laboratory of Polymer Physics and Chemistry, Changchun Institute of Applied Chemistry, Chinese Academy of Sciences, Changchun, Jilin 130022, P. R. China.
*e-mail: xzduan@ciac.ac.cn; qizhang@cuhk.edu.cn.

This file includes:

Supplementary methods

Supplementary Table 1-3

Supplementary Figures and Legends 1-23

Contents

Supplementary Information....1
Contents....2
1. Supplementary Methods....4
Mechanical tests....4
Dynamic mechanical test....4
Materials characterizations....4
Molecular dynamics simulation....5
2. Supplementary Tables....10
Supplementary Table 1 Recipes....10
Supplementary Table 2 Universal recipes....11
Supplementary Table 3 Artificial seawater....11
3. Supplementary Figures....12
Supplementary Fig. 1 Miscibility of polymer/IL system....12
Supplementary Fig. 2 Macroscopic performance of AUA tape....12
Supplementary Fig. 3 Residual adhesive....13
Supplementary Fig. 4 Swelling property and transparency....13
Supplementary Fig. 5 Dry condition....14
Supplementary Fig. 6 Repeatability....15
Supplementary Fig. 7 DSC....16
Supplementary Fig. 8 Dissipated energy....16
Supplementary Fig. 9 Stress relaxation....17
Supplementary Fig. 10 Linear and crosslinked AUA....17
Supplementary Fig. 11 Weight loss during immersion....18
Supplementary Fig. 12 Ionic liquid content....18
Supplementary Fig. 13 Ionic liquid removal....19
Supplementary Fig. 14 Universality....19
Supplementary Fig. 15 Nano-indentation test....20
Supplementary Fig. 16 Contact angle....20
Supplementary Fig. 17 Hydrophobicity of substrates....21
Supplementary Fig. 18 FTIR....21
Supplementary Fig. 19 SAXS....22
Supplementary Fig. 20 MD simulation....23

Supplementary Fig. 21 Vertical distributions after rearrangement................ 24
Supplementary Fig. 22 Environmental adaptability..25
Supplementary Fig. 23 Monomer removal... 25

1. Supplementary Methods

Mechanical tests. Mechanical properties were measured by a tension machine (UTM 6104, SUNS Technology). All the tensile tests were performed at 50 mm/min with the samples cut into rectangular strips of 10 mm in width, and 50 mm in length. For the loading-unloading test, samples were stretched to ε = 10%, followed by unloading at the same strain rate without waiting time. The dissipated energy was computed through the area enclosed by the loading-unloading curve. For the relaxation test, a fixed strain of 5% was applied to the samples, and the evolution of the stress was recorded as a function of time.

Dynamic mechanical test. The loss modulus (*G*') and storage modulus (*G*'') of AUA were determined by a dynamic mechanical analyser (Q850, TA Instruments) at 25 °C in tensile mode. The frequency ranges from 0.1 to 100 Hz and the amplitude was fixed at 0.5% strain.

Materials characterizations. Thermogravimetric analysis (TGA) was performed by a TGA55 system under a nitrogen flow at a heating rate of 10 °C/min. Differential scanning calorimetry (DSC) measurement was carried out using a calorimeter (DSC 2500, TA Instruments) under a nitrogen flow, where the samples were placed in alumina crucibles (flow rate: 50 mL·min^{-1}; temperature range: −80 to 80 °C; heating rate: 10 °C·min^{-1}). The indentation

force curves of AUA surfaces were recorded on an nano-indentor (Hysitron TI 980 TriboIndenter, Bruker) equipped with a standard berkovich probe. The loading rate is 1.2 mN/s and the peak force is 6 mN. Contact angle measurement was conducted with an OCA25 (Data Physics, Germany) equipped with an ES-N 10 syringe. Attenuated total reflectance-Fourier transform infrared (ATR−IR) spectrum was recorded using a Nicolet iS10 instrument (Thermo Fisher Scientific) equipped with a Smart iTR accessory. The small-angle X-ray scattering (SAXS) profiles were measured by Xeuss 2.0 (Xenocs) at a wavelength of 0.154 nm (SDD: 1185 mm; exposure time: 300 s).

Molecular dynamics simulation. We employ the coarse-grained Molecular Dynamics (MD) simulation to explore the structural and thermodynamic properties of the polymer/IL complexes and the interface effects on the redistributions of different species within the complexes.

In the simulation, we model each copolymer as a linear freely-jointed chain composed of (hydrophobic) BzMA beads and (hydrophilic) HEMA beads. The chain length of each polymer is set as N_p = 100 and the sequence of BzMA and HEMA beads is randomly set. We model the cations and anions of the ionic liquids, the crosslinkers, and water molecules as independent spherical beads, with each cation bead or anion bead carrying a positive charge (+1*e*) or a negative charge (-1*e*) at the center. The BzMA beads, HEMA beads, cations, anions, crosslinkers and water molecules are classified as nonpolar, polar,

charged, charged, nonpolar and polar beads, respectively. For simplicity, we assume that all aforementioned beads have the same diameters σ = 1.0 (which corresponds to 4 Å). The *van der Waals* interactions between these beads are modeled using Lennard-Jones (LJ) potential,

$$U_{LJ}(r) = \begin{cases} 4\varepsilon_{\mathrm{LJ}}\left[\left(\frac{\sigma}{r}\right)^{12} - \left(\frac{\sigma}{r}\right)^{6} + S\right], r \le r_c \\ 0, r > r_c \end{cases} \quad (1)$$

Herein, we change the energetic parameter as $\varepsilon_{\mathrm{LJ}}$ = 1.0 ~ 3.0 (which corresponds to 1.0 ~ 3 $k_{\mathrm{B}}T$ at room temperature, T = 298 K) and set the cutoff to be $r_{\mathrm{c}} = 2^{1/6}\sigma \sim 2.5\sigma$, to account for inter-molecular interactions (such as volume excluded effect, hydrophobic and hydrophilic interactions, and solvation interactions, etc.) between these nonpolar, polar and charged beads. The LJ potential is shifted to 0 at r_c by S. We use σ = 1.0 (which corresponds to 4 Å) and $\varepsilon_{\mathrm{LJ}}^{0}$ = 1.0 (which corresponds $k_{\mathrm{B}}T$ at room temperature) as the length and energy units. We apply Coulomb potential to account for the electrostatic interactions between charged species,

$$U_{\mathrm{Coul}} = \frac{Z_1 Z_2 e^2}{4\pi\varepsilon_0\varepsilon_{\mathrm{r}} r} \quad (2)$$

where Z_1 and Z_2 indicate the valences of the charged beads separated in a distance r, ε_0 represents the vacuum permittivity and ε_{r} = 10 is the dielectric constant of the system. The U_{Coul} is calculated using the particle-particle-particle-mesh (PPPM) algorithm with an accuracy of 10^{-5}. Further, we model the bond effects between the connected monomer beads in polymers through the finitely extensible nonlinear elastic (FENE) potential,

$$U_{\mathrm{FENE}}(r_b) = -\frac{1}{2}kR_0^2 \ln\left[1-\left(\frac{r_b}{R_0}\right)^2\right] \quad (3)$$

where k = $30\varepsilon_{\mathrm{LJ}}^0/\sigma^2$ represents the spring constant and R_0 = 1.5 σ is the maximum bond extension.

The simulations are performed within the canonical (NVT) ensemble via the Large-scale Atomic/Molecular Massively Parallel Simulator (LAMMPS) package. We set the integration MD time step as 0.002 (which corresponds to 4 fs) and control the temperature as 298K via the Langevin thermostat. In all these simulations, the total number density of the beads is set as ρ ~ 0.85, which corresponds to the typical liquid states in simulations. In the study of bulk polymer/IL mixtures at as-prepared and hydrated states, we randomly put all species in a cubic simulation box of L = 20σ (8 nm) with the 3D periodic boundary conditions. For the polymer/IL complex at the as-prepared state, the ratio between BzMA beads, HEMA beads, cations, anions and crosslinkers is set as 1913:2587:1145:1145:10, and for the polymer/IL complex at the hydrated state, the ratio between BzMA beads, HEMA beads, cations, anions and crosslinkers and water molecules is set as 1403:1897:838:838:6:1818, both of which are consistent with our experiments. In the study of interface effect on polymer/IL complexes, we randomly put the hydrated polymer/IL complex within the range of 2 nm (5σ) < z < 10 nm (25σ) in a rectangular simulation box of L×L×H = 20σ×20σ×30σ = 8nm×8nm×12nm, where L is the box length in the x- and y- directions and H denotes the box length in the z-direction, and periodic boundary conditions are applied in the x- and y-

directions. For the case of polymer/IL complex in between double water phases, we randomly generate n_w = 1700 water beads in the region of 0 nm (0σ) < z < 2 nm (5σ) and the same amount of water beads in the region of 10 nm (25σ) < z < 12 nm (30σ) to model the water phases. We add two virtual flat walls (with each one composed of 20×20 beads) at z = 2 nm and z = 10 nm to hinder the interpenetration between the polymer/IL complex and the water phase. For the polymer/IL complex in between a water interface and a hydrophobic PET substrate, we randomly generate n_w = 1700 water beads in the range of 10 nm (25σ) < z < 12 nm (30σ) to simulate the water phase, and regularly generate 20×20×6 fixed hydrophobic PET beads closely packed in the range of 0 nm (0σ) < z < 2 nm (5σ) to model the hydrophobic substrate. Herein, the hydrophobic PET beads are treated as the apolar beads. Similarly, a virtual flat wall (with each one composed of 20×20 beads) is put at z = 10 nm to hinder the overflow of complex species into the water phase. In the simulations, we first use 10^6 MD steps to obtain the equilibrated configurations of the complexes (within the scope of 2nm < z < 10nm) and water beads, after which we eliminate the interactions between the walls and water beads from the water phase and use 10^6 MD steps to account for the interactions between the water phase and the polymer/IL complexes. Further, we use 10^6 MD steps as the production runs. For all the cases, including polymer/IL complex at as-prepared and hydrated states, in between the double water phases, and in between a water phase and a hydrophobic PET substrate, we perform 20

simulations with different initial configurations and obtain the final results.

2. Supplementary Tables

Supplementary Table 1. The recipes of precursor solutions for the synthesis of AUA tapes.

AUA-*f*-*C*-*I*	BzMA (g)	HEMA (g)	EMITFSI (g)	DVB (μL)	PI-184 (g)
AUA-0.0-0.15-40	3.00	0.00	2.00	6.00	0.03
AUA-0.2-0.15-40	2.40	0.60	2.00	6.00	0.03
AUA-0.3-0.15-40	2.10	0.90	2.00	6.00	0.03
AUA-0.4-0.15-40	1.80	1.20	2.00	6.00	0.03
AUA-0.5-0.15-40	1.50	1.50	2.00	6.00	0.03
AUA-0.6-0.15-40	1.20	1.80	2.00	6.00	0.03
AUA-0.7-0.15-40	0.90	2.10	2.00	6.00	0.03
AUA-0.5-0.15-0	1.50	1.50	0.00	6.00	0.03
AUA-0.5-0.15-30	1.50	1.50	1.28	6.00	0.03
AUA-0.5-0.15-35	1.50	1.50	1.62	6.00	0.03
AUA-0.5-0.15-45	1.50	1.50	2.45	6.00	0.03
AUA-0.5-0.15-50	1.50	1.50	3.00	6.00	0.03
AUA-0.5-0.00-40	1.50	1.50	2.00	0.00	0.03
AUA-0.5-0.40-40	1.50	1.50	2.00	16.00	0.03
AUA-0.5-1.00-40	1.50	1.50	2.00	40.00	0.03

Supplementary Table 2. The recipes of precursor solutions for the synthesis of P(BzMA-co-DMAA)/EMITFSI, P(EMA-co-HEMA)/EMITFSI and P(BzMA-co-HEMA)/$BMIPF_6$ ionogels.

	BzMA/ EMA (g)	HEMA/ DMAA (g)	EMITFSI/ $BMIPF_6$ (g)	DVB (μL)	PI-184 (g)
P(BzMA-co-DMAA)/EMITFSI	1.80	1.20	2.00	6.00	0.03
P(EMA-co-HEMA)/EMITFSI	1.50	1.50	2.00	6.00	0.03
P(BzMA-co-HEMA)/$BMIPF_6$	1.80	1.20	2.00	12.00	0.03

Supplementary Table 3. The recipe of artificial seawater.

NaCl ($g \cdot L^{-1}$)	$CaCl_2$ ($g \cdot L^{-1}$)	$MgCl_2$ ($g \cdot L^{-1}$)	KCl ($g \cdot L^{-1}$)	$MgSO_4$ ($g \cdot L^{-1}$)	$NaHCO_3$ ($g \cdot L^{-1}$)
26.73	1.15	2.26	0.72	3.25	0.20

3. Supplementary Figures

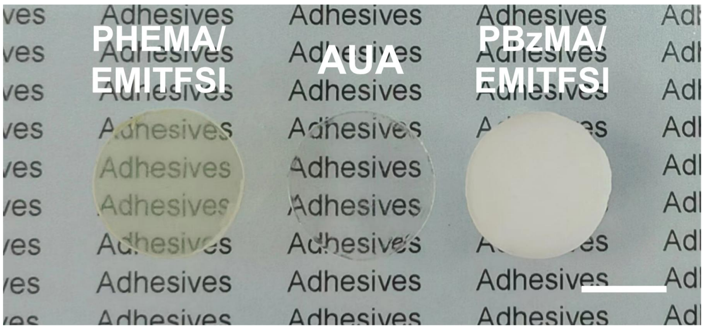

Supplementary Fig. 1 Miscibility of polymer/IL system. Photographs of as-prepared PHEMA/EMITFSI gel, PBzMA/EMITFSI composite and AUA (*f* = 0.5), comparing their transparency (scale bar = 10 mm).

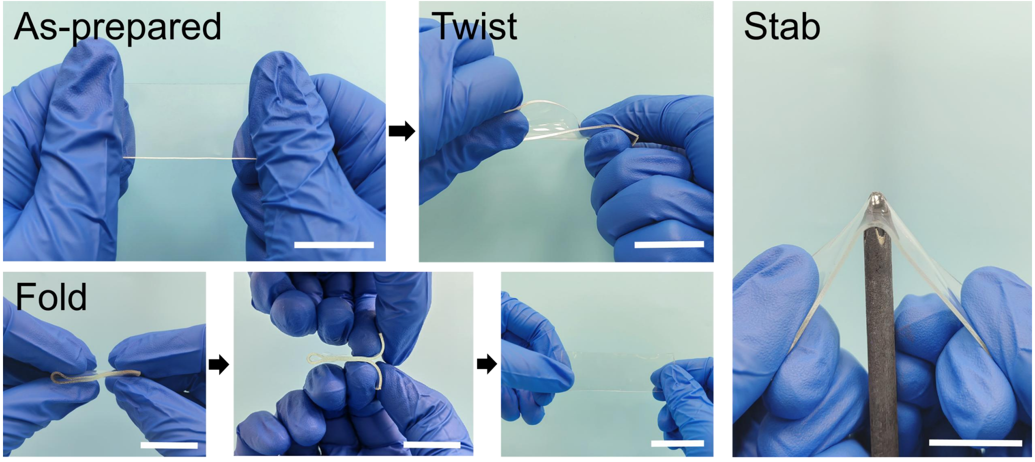

Supplementary Fig. 2 Macroscopic performance of AUA tape. Photographs showing the mechanical performance of as-prepared AUA tape under different actions including twisting, folding and stabbing (scale bar = 20 mm), exhibiting its robustness and flexibility.

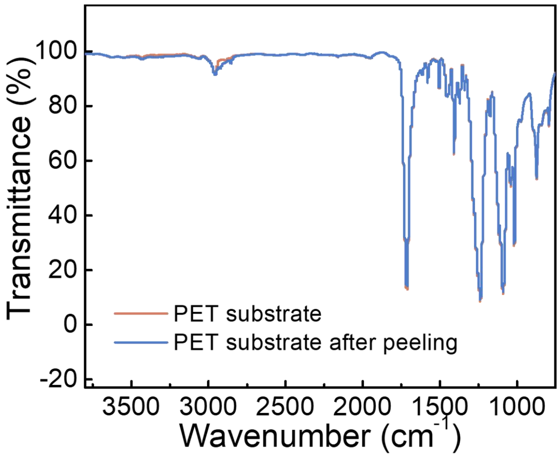

Supplementary Fig. 3 Residual adhesive. The FT-IR spectra of the PET substrate, comparing the pristine substrate with the substrate after attaching by AUA material, water-immersing and peeling, showing no residual adhesive after peeling.

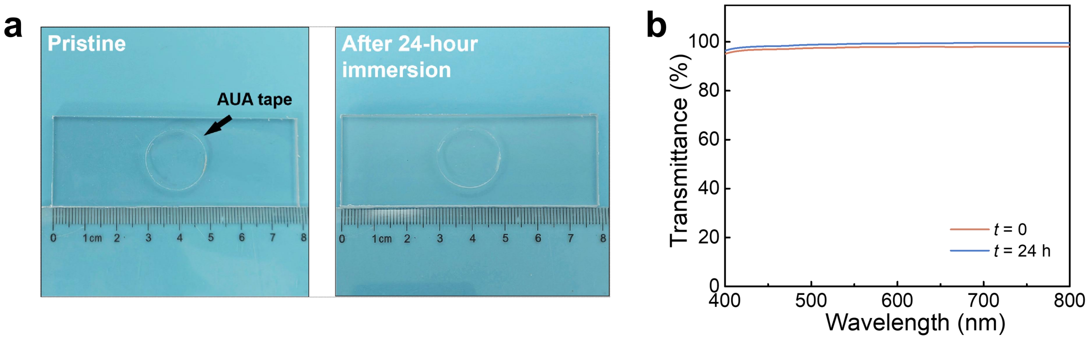

Supplementary Fig. 4 Swelling property and transparency. a Photographs of the AUA tape at t = 0 and t = 24 h, cut into d = 19 mm disk, showing no size change or swelling during the immersion. **b** The transmittance spectrum of AUA at t = 0 and t = 24 h, showing no transparency difference between before and after immersion.

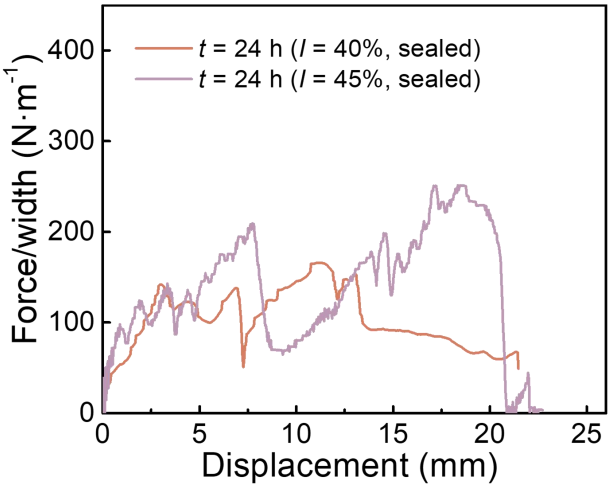

Supplementary Fig. 5 Dry condition. Force-displacement curves recorded by 90-degree peeling tests of AUA tapes at *I* = 40% and *I* = 45% on PET substrates. The post-adhere substrate was carefully sealed in a plastic ziplock bag for 24 hours, which was to provide a dry ambient for comparison.

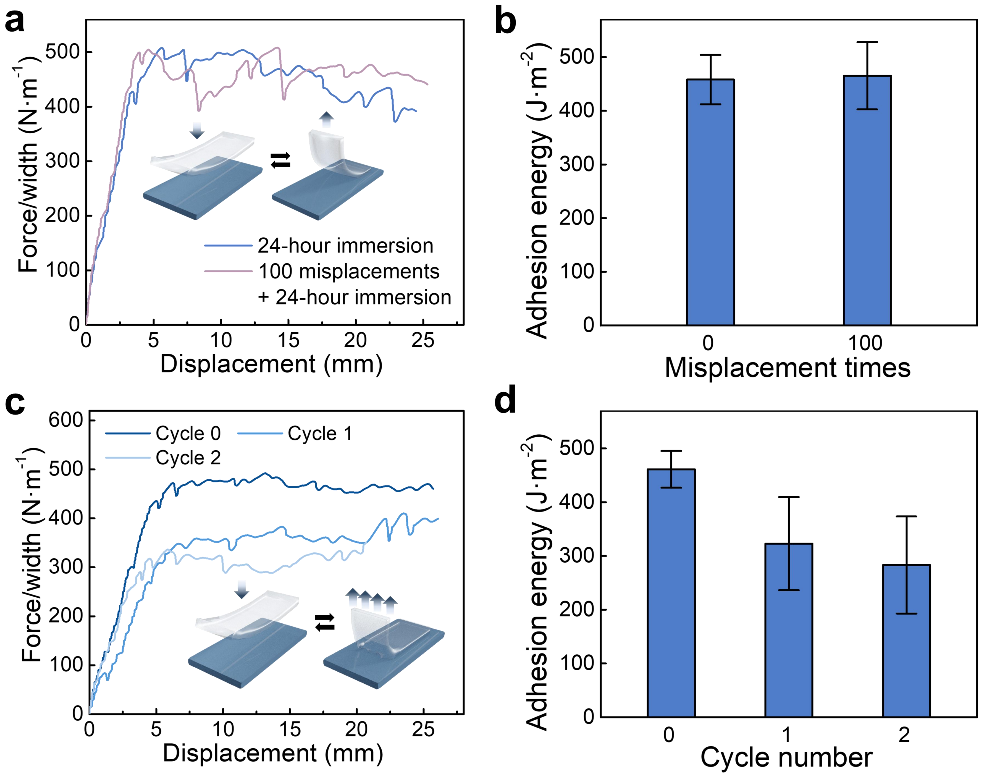

Supplementary Fig. 6 Repeatability. The reusability of AUA tapes. **a** Force-displacement curves and **b** the corresponding interfacial toughness of AUA tapes at t = 24 h after 100 misplacements at t = 0 compared with the pristine. **c** Force-displacement curves and **d** the corresponding interfacial toughness of AUA tapes at t = 24 h during 2 immerse-peel-dry cycles. Values represent the mean and standard deviation, where n = 3.

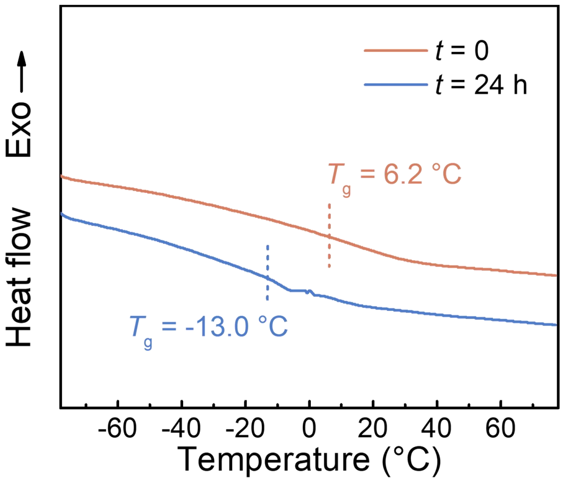

Supplementary Fig. 7 DSC. DSC curves of AUA comparing *t* = 0 and *t* = 24 h. The glass transition temperature of AUA shifted from 6.2 °C to -13.0 °C after plasticized (water-immersion).

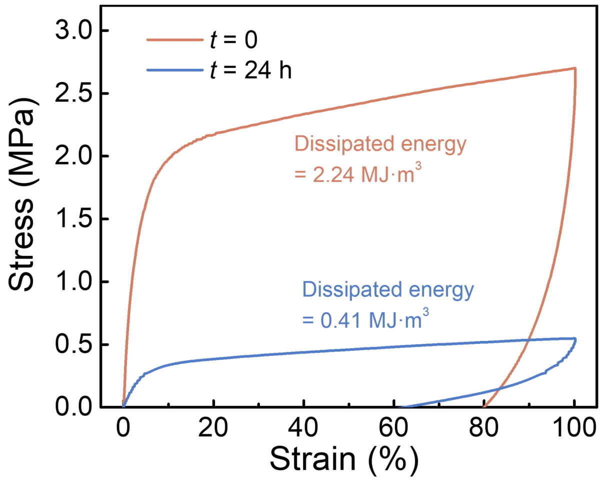

Supplementary Fig. 8 Dissipated energy. Typical loading-unloading curves of AUA comparing *t* = 0 and *t* = 24 h and the corresponding dissipation energy. The dissipated energy was computed through the area enclosed by the loading-unloading curve.

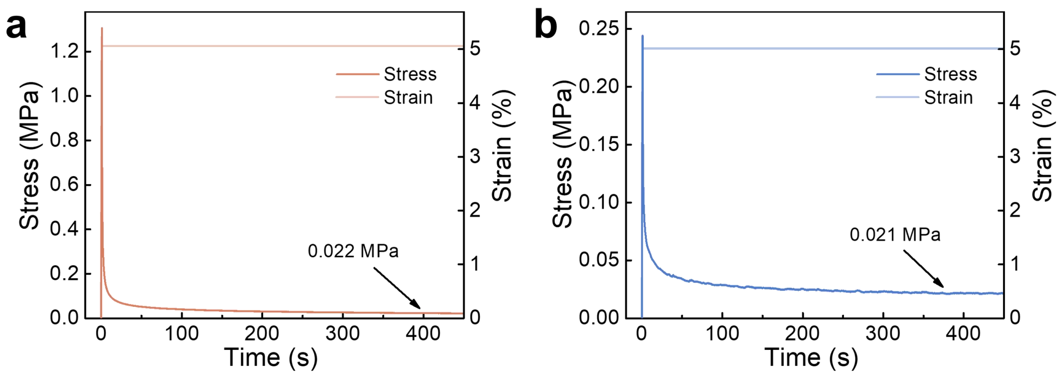

Supplementary Fig. 9 Stress relaxation. The evolution of stress for the AUA at **a** $t = 0$ and **b** $t = 24$ h during a relaxation test with a fixed strain of 5%.

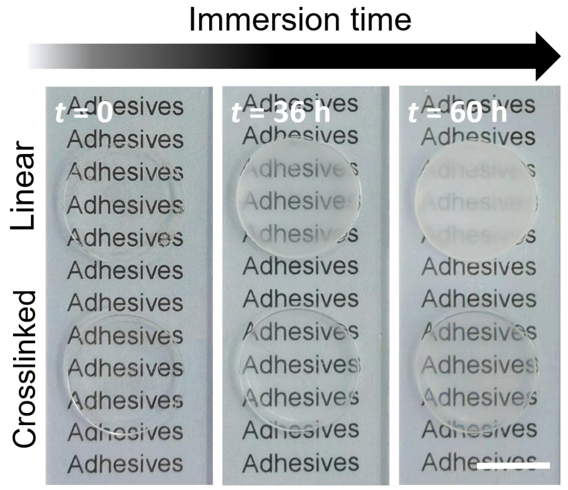

Supplementary Fig. 10 Linear and crosslinked AUA. Photographs of linear and crosslinked AUA (scale bar = 10 mm). For the linear AUA, the opaque region started propagating from the rim of the sample after 36-hour immersion and the whole sample turned opaque in 60 hours. In contrast, the crosslinked AUA showed less transparency change during immersion.

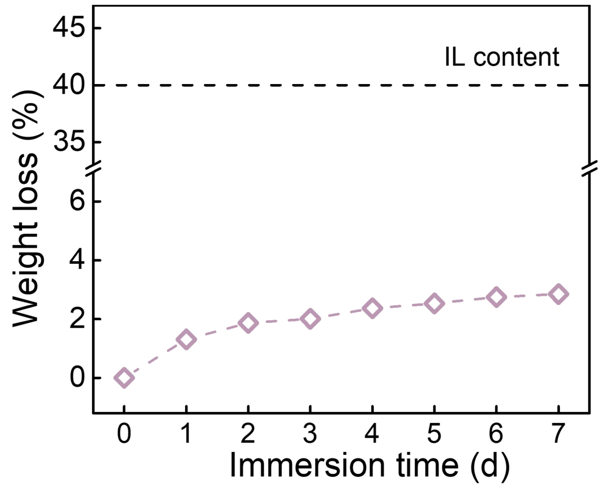

Supplementary Fig. 11 Weight loss during immersion. Weight loss of the bulk AUA material immersion recorded by weighing at different immersion time. The tested samples were obtained after being immersed in DI water for 1-7 days and then dried in a 110 °C oven until reached weight equilibrium.

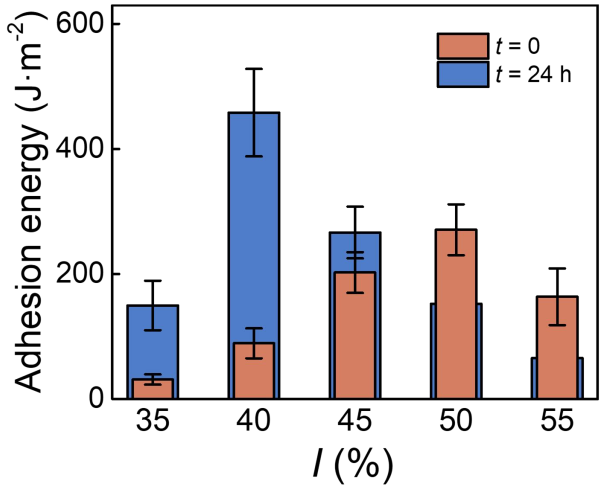

Supplementary Fig. 12 Ionic liquid content. Adhesion energy comparing t = 0 and t = 24 h at different ionic liquid content (I). Values represent the mean and standard deviation, where n = 3.

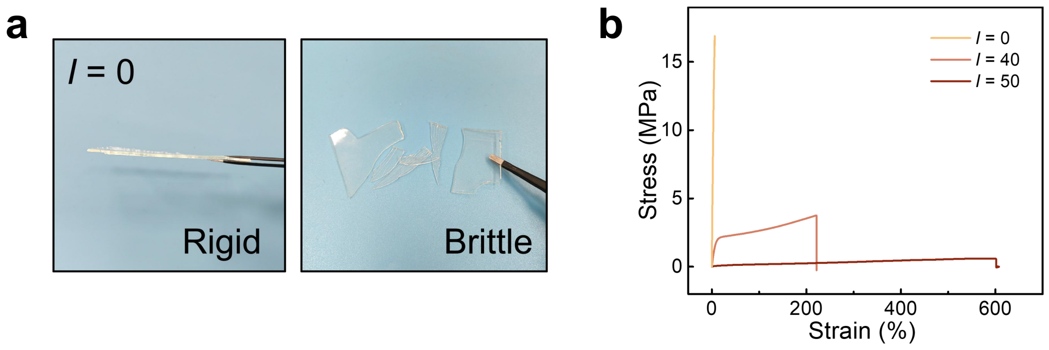

Supplementary Fig. 13 Ionic liquid removal. **a** Photographs showing the rigidity and brittleness when the ionic liquid was removed. **b** Stress-strain curves of AUA at different *I*.

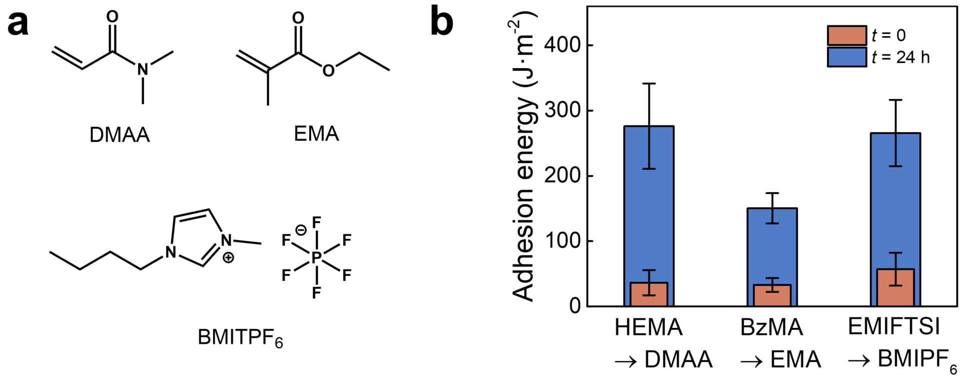

Supplementary Fig. 14 Universality. A universal approach to AUA. **a** Substitutions of hydrophilic monomer (DMAA), hydrophobic monomer (EMA) and ionic liquid ($BMIPF_6$) used in the universal method, and **b** the corresponding adhesion results. Values represent the mean and standard deviation, where n = 3.

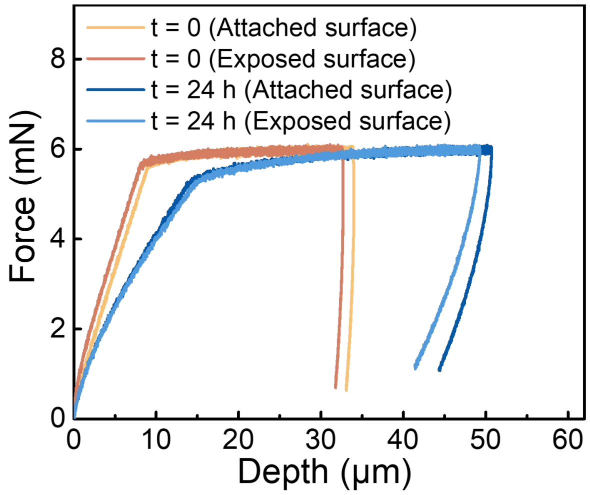

Supplementary Fig. 15 Nano-indentation test. The loading-holding-unloading force curve of AUA surfaces during the nano-indentation tests. The loading rate is 1.2 mN/s, the peak force is 6 mN, and the holding time was prolonged to 60 s to eliminate the hysteresis and creep of the dissipated matrix.

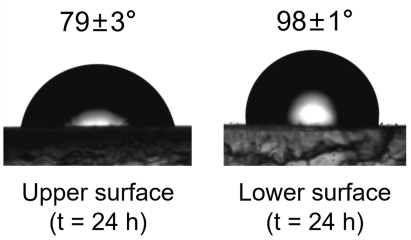

Supplementary Fig. 16 Contact angle. Contact angle of water on the two sides of AUA (t = 24 h), using glass as substrate during immersion.

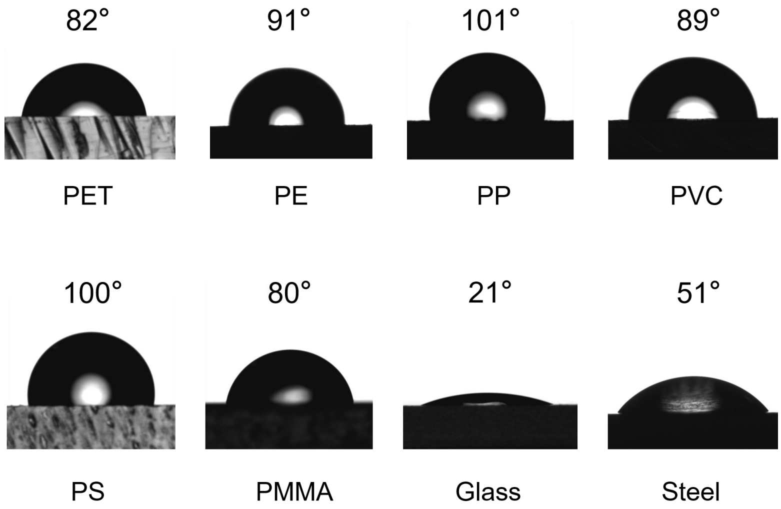

Supplementary Fig. 17 Hydrophobicity of substrates. Contact angle of water on the tested substrates.

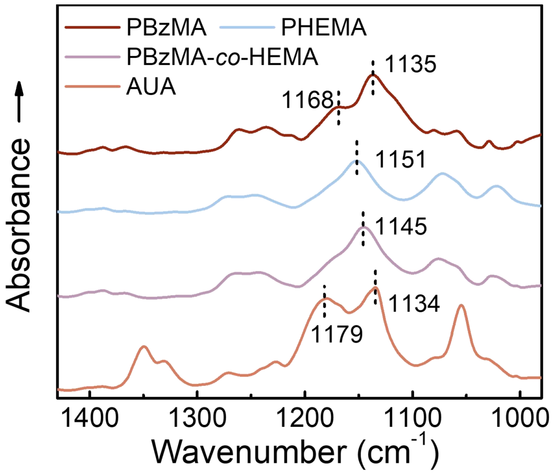

Supplementary Fig. 18 FTIR. ATR-FTIR spectra showing the stretching region of C-O-C in PBzMA, PHEMA, PBzMA-co-HEMA and AUA, respectively.

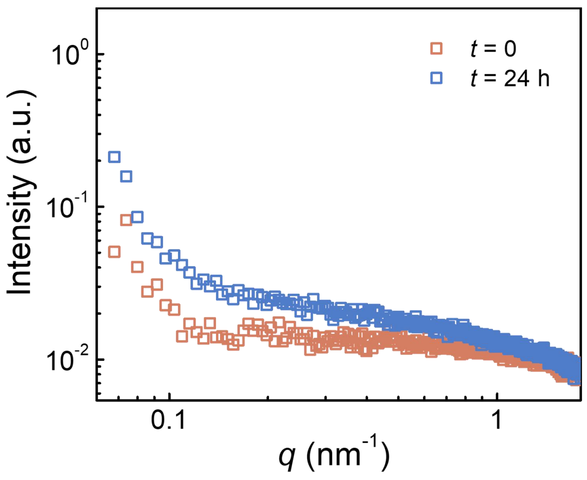

Supplementary Fig. 19 SAXS. SAXS profiles of AUA comparing *t* = 0 and *t* = 24 h.

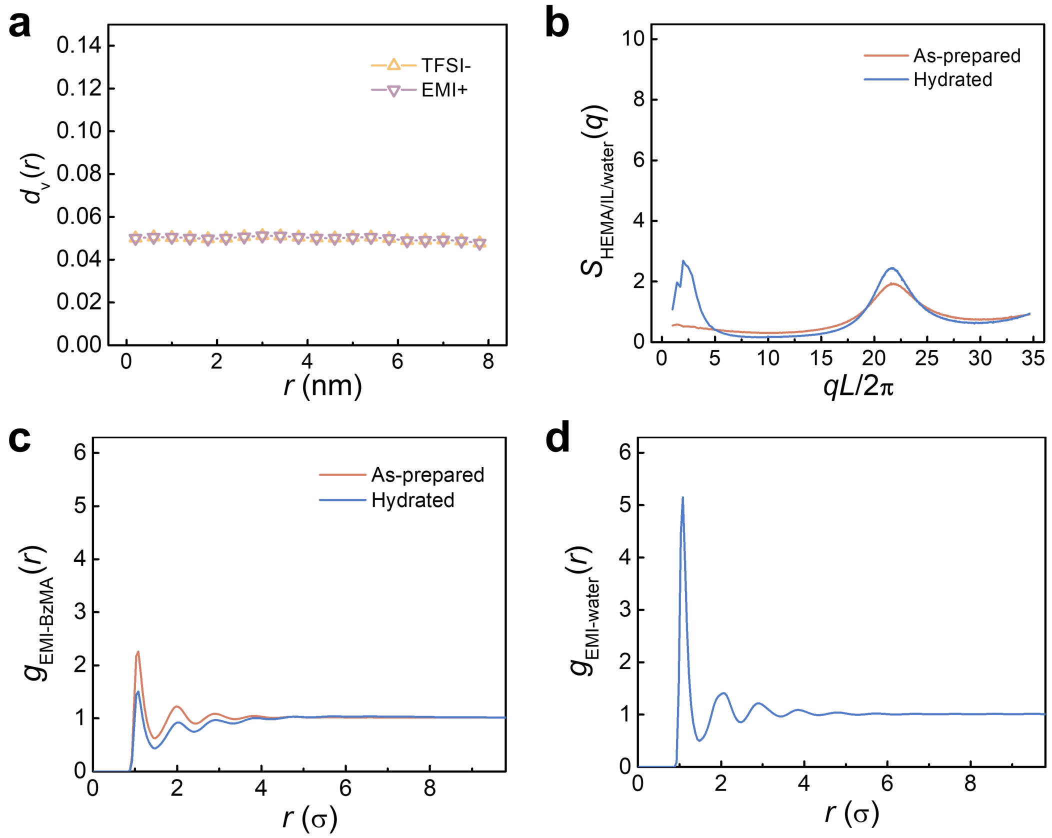

Supplementary Fig. 20 Static simulation. **a** Vertical distributions [$d_v(r)$] of $TFSI^-/EMI^+$ ions in polymer/IL complex at as-prepared state. **b** Static structure factor for HEMA/IL/water [$S_{HEMA/IL/water}(q)$] in polymer/IL complex at as-prepared and hydrated states. Radial distribution functions of **c** BzMA beads [$g_{EMI\text{-}BzMA}(r)$] and **d** water beads [$g_{EMI\text{-}water}(r)$] around each EMI^+ ion in polymer/IL complex at as-prepared and hydrated states.

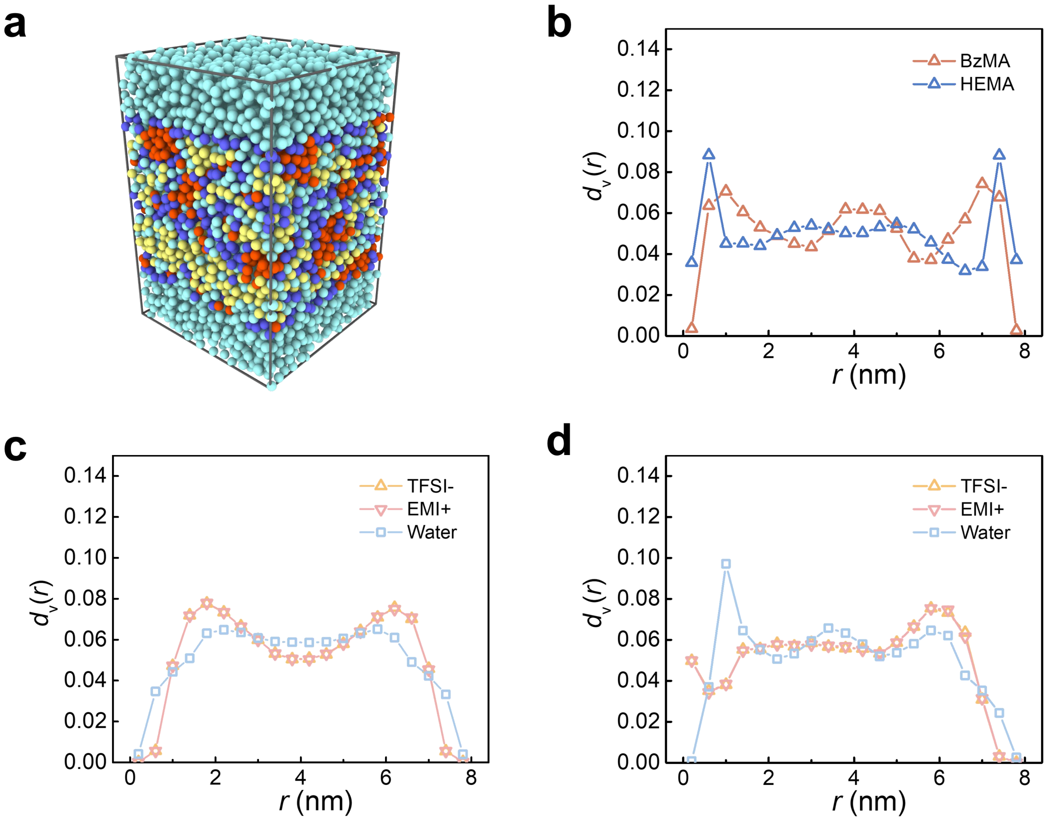

Supplementary Fig. 21 Vertical distributions after rearrangement. a Simulation snapshots for hydrated polymer/IL complex between two (upper and lower) water phases. Vertical distributions [$d_v(r)$] of **b** BzMA/HEMA and **c** $TFSI^-$/EMI^+/water in hydrated polymer/IL complex between two water phases (r = 0 nm indicates the interface between the lower water phase and the polymer/IL complex). **d** Vertical distributions [$d_v(r)$] of $TFSI^-$/EMI^+/water in hydrated polymer/IL complex between an upper water phase and a lower hydrophobic substrate (r = 0 nm indicates the interface between the polymer/IL complex and the substrate).

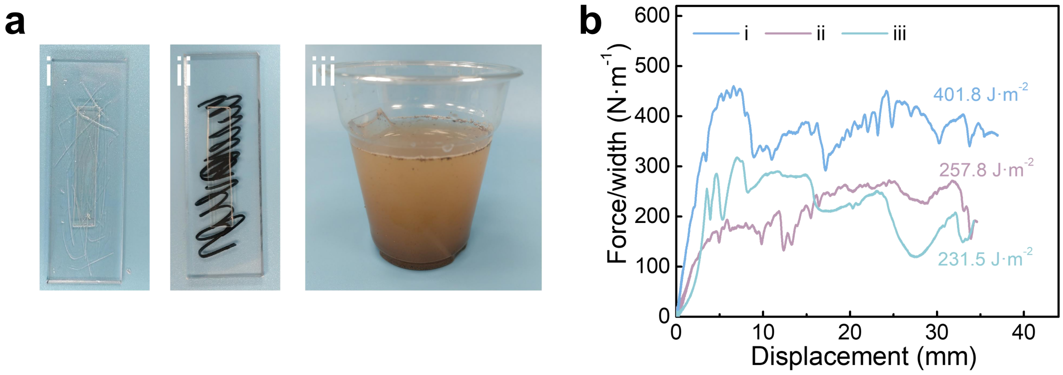

Supplementary Fig. 22 Environmental adaptability. a Photographs of three 3 types of simulative natural conditions: underwater scraped surface, underwater oil painted surface and muddy aqueous environment, respectively (substrate: PET). **b** The corresponding force-displacement curves of AUA during 90-degree peeling on these conditions.

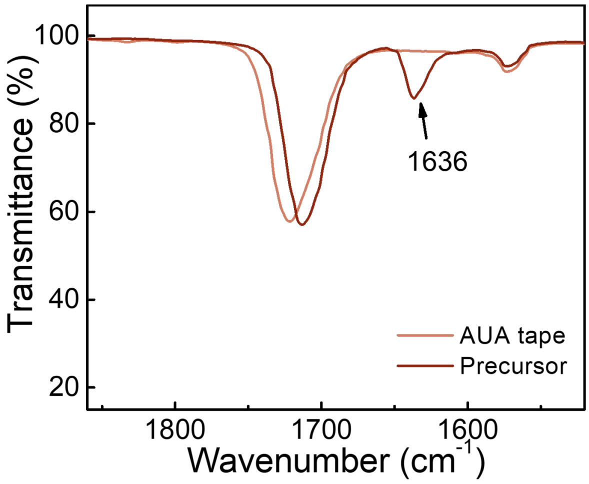

Supplementary Fig. 23 Monomer removal. The FT-IR spectra comparing the AUA tape and its precursor. The characteristic peak located at 1636 cm^{-1} is assigned to C=C stretching vibration, which disappeared after photocuring and vacuumed, thus indicating the absence of residual monomers.
